# The impact of body mass index on laboratory, clinical outcomes and treatment costs in assisted reproduction: a retrospective cohort study

**DOI:** 10.1186/s12905-022-02036-x

**Published:** 2022-11-28

**Authors:** Victoria Campos Dornelles, Marta Ribeiro Hentschke, Mariangela Badalotti, Isadora Badalotti Telöken, Vanessa Devens Trindade, Bibiana Cunegatto, Natália Fontoura de Vasconcelos, Bartira Ercília Pinheiro da Costa, Alvaro Petracco, Alexandre Vontobel Padoin

**Affiliations:** 1 Fertilitat - Reproductive Medicine Center, Rua Gomes Jardim, 201 Torre Norte 15º andar - Santana, Porto Alegre, Rio Grande do Sul 91530-001 Brazil; 2 Graduate Program in Medicine and Health Sciences - School of Medicine, Pontifical Catholic University of Rio Grande do Sul, PUCRS, Av. Ipiranga, 6681, Prédio 12A Partenon, Porto Alegre, Rio Grande do Sul 90619-900 Brazil

**Keywords:** Overweight, Obesity, Infertility, In Vitro Fertilization, Costs and Costs Analysis

## Abstract

**Background:**

The aim of this study was to evaluate the influence of the body mass index (BMI) on laboratory, clinical outcomes and treatment costs of assisted reproduction, as there are still controversial and inconclusive studies on this subject.

**Methods:**

This research was retrospective cohort study, including women undergoing assisted reproduction in a Reproductive Medicine Center between 2013 and 2020. The participants were divided into groups according to BMI (kg/m^2^): Group 1 < 25; Group 2, 25–29.9 and Group 3, ≥ 30. A total of 1753 in vitro fertilization (IVF) fresh embryo transfer (ET) cycles were included for assisted reproduction outcomes analysis and 1869 IVF-ET plus frozen embryo transfer (FET) for cumulative pregnancy analysis.

**Results:**

As higher the BMI, higher was the proportion of canceled IVF cycles (G1 (6.9%) vs. G2 (7.8%) vs. G3 (10.4%), *p* = 0.002) and gonadotropin’s total dose (IU) and treatment costs (G1 (1685 ± 595, U$ 683,02) vs. G2 (1779 ± 610, U$ 721,13) vs. G3 (1805 ± 563, U$ 764,09), *p* = 0.001). A greater number of mature oocytes was observed in G1 and G2 (6 [6.4–7.0] vs. 6 [5.6–6.6] vs. 4 [4.6–6.7], *p* = 0.011), which was not found in oocyte maturity rate (*p* = 0.877). A significant linear tendency (*p* = 0.042) was found in cumulative pregnancy rates, pointing to worse clinical outcomes in overweight and obese patients.

**Conclusion:**

These findings highlight the importance of considering the higher treatment costs for these patients, beyond all the well-known risks regarding weight excess, fertility, and pregnancy, before starting IVF treatments.

## Background

Obesity and infertility are in growing prevalence in the world, being considered as concerning public health conditions [1–4]. Also, the weight excess has a well-known negative impact on female fertility, mainly related to ovulation disorders [5].

In assisted reproduction, the needs for higher doses of gonadotropins during ovarian controlled stimulation is well established for obese patients in the literature, with a direct association between these group of patients and in vitro fertilization (IVF) canceled cycles [6–8].

Despite the expected effect of obesity in oocyte quality, studies on laboratory outcomes after assisted reproduction techniques (ART) remains controversial. Some studies found significantly lower numbers of mature oocytes retrieved and fertilization rates, which is a known oocyte quality marker, while others have not found differences [9].

Also, it is still not clear whether the weight effect in women’s fertility is translated into worse clinical outcomes after embryo transfer (ET), with most of the studies analyzing frozen ET cycles (FET) in association with IVF-ET cycles. Only a few studies considered the possibility of bias since FET cycles are associated with better outcomes when compared to fresh cycles [10–13].

Therefore, assisted reproduction treatment costs are expected to be higher in overweight and obese women; however, this is also still inconclusive in the literature, with some studies having found association while others have not [14–16].

Thus, the aim of this study was to analyze whether the laboratory and clinical outcomes of assisted reproduction are influenced by the BMI’s categories, considering also the treatment costs. The main hypothesis was there is a negative effect from BMI on these outcomes.

## Methods

The present study performed a retrospective cohort study in a reproductive medicine center in the south of Brazil, which receives patients from the country’s south and southeast as it is considered one of the major region’s reproductive centers, performing about one thousand cycles per year. All data were collected from electronic records from 2013 to 2020.

This manuscript was structured following STROBE (Strengthening the Reporting of Observational Studies in Epidemiology) [17].

### Study Population

#### Inclusion criteria

Women undergoing follicle stimulation cycles at the reproductive medicine center which had weight and height registered in electronic records. The population included were mostly Caucasian women with complete high school education and considered to be in a social economical class capable of affording the high costs related to assisted reproduction in Brazil. Some of these patients performed more than one ART cycle and each one was considered as a new patient for statistical analysis and that’s why this study’s population is based on ART cycles and not patients.

#### Exclusion criteria

Women with previous chemotherapy or radiotherapy and with previous oophorectomy were excluded from the present study’s analysis. Also, patients older than 40 years old were not considered, as well as patients whose embryos were biopsied before implantation to avoid bias in the analysis of clinical outcomes.

### Sample groups

**Fig. 1:**
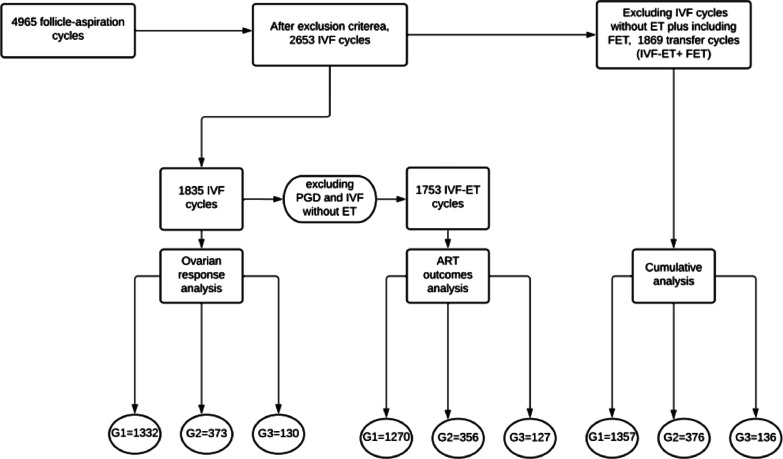
Sample’s distribution. Legend. G1. Group 1 BMI ≤ 24.9 Kg/m^2^, G2. Group 2 BMI 25–29.9 Kg/m^2^, G3. Group 3 BMI ≥ 30 Kg/m^2^. IVF. In vitro fertilization, ET. Embryo transfer, PGD. Embryonic biopsy, ART. Assisted reproduction techniques. For groups division, WHO, 2000.(1)

Each analysis was made by dividing samples into three groups according to the BMI, in line with WHO classification [2] (Group 1: BMI ≤ 24.9 kg/m^2^, Group 2: BMI 25–29.9 kg/m^2^, Group 3: BMI > 30 kg/m^2^), as shown in Fig. 1.

A total of 4965 follicle stimulation cycles were initially included. After applying exclusion criteria, 2653 IVF cycles were included and divided according to BMI. The analysis was performed into two steps. In the first one, the ovarian stimulation outcomes (laboratory outcomes) were compared between groups. For this analysis, it was taken into consideration individual ovarian stimulation protocol adjusted by infertility causes and it was also analyzed the canceled cycles rate. For ovarian response analysis, the IVF canceled cycles were excluded since patients have not completed it, resulting in 1835 IVF cycles.

In the second step, ART clinical outcomes were analyzed, when fresh embryo transfer (ET) cycles were performed, totaling 1753 IVF-ET cycles.

FET cycles were included into second step analysis only for cumulative pregnancy rate calculation, resulting in 1869 ET cycles (IVF-ET fresh cycles plus FET-ET cycles).

It was also performed an analysis to evaluate the impact of female obesity on ART costs. Evaluating the average gonadotropin costs in Brazil, each IU of menotropin (hMG) is U$ 0.32, and the average recombinant FSH (FSHr) and FSHr + LHr costs per IU are U$ 0.44. Then, the average value of these three gonadotropins is U$ 0.40/IU.

### Definitions

All laboratory definitions followed Vienna Consensus [18]. Biochemical pregnancy was defined as increased serum human chorionic gonadotropin (hCG) concentrations, and clinical pregnancy was defined by the presence of one or more intrauterine gestational sacs confirmed by an ultrasound image from 5 weeks of gestational age. Cumulative pregnancy rate was defined as the number of pregnancies considering all ET cycles from each patient.

### Controlled ovarian stimulation protocol

The controlled ovarian stimulation was performed with gonadotropin-releasing hormone (GnRH) agonist or antagonist protocols and gonadotropins (75–300 IU/daily) adjusted according to each patient’s response and selected considering individual clinical indications. The gonadotropin choice was made among corifollitropin alfa, recombinant follicle-stimulating hormone (FSHr), human urinary gonadotropin (hMG), combination of recombinant FSH and luteinizing hormone (LHr) and eventually an association was indicated. The trigger was performed by recombinant hCG 250 mcg or 0,2 mg triptorelin (GnRH agonist) 34–36 hours before ultrasonography-guided oocyte retrieval, according to each patient’s indications, when three or more follicles reached 17 mm, or one follicle achieved 20 mm. The oocyte retrieval was performed, and the oocyte maturity was analyzed. Intracytoplasmic Sperm Injection (ICSI) was the technique used in the embryo laboratory for all fertilization. The embryos were transferred on days 2–6 of development. The luteal phase was supported using 600–800 mg of intravaginal micronized progesterone per day.

The spare embryos (not transferred) were vitrified and, depending on the clinical outcome, were transferred at appropriate time. The frozen technique followed a unique laboratory vitrification protocol. For FET-ET the endometrial preparation was made with oral or transdermal oestrogen.

For pregnancy diagnosis, serum beta-hCG was collected after 10–12 days of ET, and an obstetric ultrasound was performed after 2 weeks of hCG result. After clinical pregnancy confirmation, patients were referred to prenatal care with their chosen obstetrician.

### Statistical analysis

Social Package for Social Sciences (SPSS) for Windows, version 22.0 (SPSS IncChicago, IL, USA) was used for statistical analysis. Data were expressed as median with interquartile range (IR) when not normally distributed. When normally distributed, it was expressed as mean ± standard deviation (SD) or frequency (%) when appropriated. Continuous variables were compared using ANOVA, categorical variables with the Chi-square test and nonparametric with U Mann-Whitney or Kruskal-Wallis tests when appropriated. Multiple logistic regression and post-hoc curve analysis were performed on groups comparison. To consider patients and the number of cycles for these analyses, generalized estimating equations (GEE) was performed. Statistical significance was defined as *p* < 0.05.

### Ethics

Research Ethics Committee of Pontifical Catholic University of Rio Grande do Sul (n^o^ protocol 4.085.223; CAAE 78763917.5.0000.5336) approved this study and waived the informed consent term for participants. All authors signed a data compromise and confidentiality responsibility term before collecting data. Declaration of Helsinki guidelines and regulations were considered for all methodology performed.

## Results

The mean maternal age was 35.5 ± 3.6 years old (yo) and the mean BMI (kg/m^2^) was 23.6 ± 3.7 in the whole sample. When comparing groups 1, 2 and 3, respectively, the following results regarding maternal age and BMI were found: 35.5 ± 3.6 vs. 35.9 ± 3.6 vs. 35.0 ± 4.3 (*p* = 0.040) and 21.7 ± 1.7 vs. 26.8 ± 1.3 vs. 32.9 ± 2.3 (p < 0.05). Other baseline characteristics, such as paternal age and infertility diagnosis, are presented in Table 1. Endometriosis was found as infertility diagnosis in 19.8% from the sample and when comparing groups 1, 2 and 3 the following results were respectively found: 22% vs. 14.8% vs. 11.4% (*p* = 0.001).

**Table 1:** Baseline characteristics comparing groups according to BMI

Characteristics	G1 *n* = 1660	G2 *n* = 459	G3 *n* = 177	Total	*p*
Maternal Age (years)	35.5 ± 3.6	35.9 ± 3.6 *	35 ± 4.3	35.5 ± 3.6	0.040^a, b^
Paternal Age (years)	38.4 ± 6.2	38.7 ± 6	39 ± 8.1	38.5 ± 6.3	0.581^a, b^
Infertility diagnosis (all %)					
Male factor	33.2	41 *	38.6	35.1	0.016^c^
Ovarian factor	16.7	18.2	22.8	17.4	0.215^c^
Tubal factor	18.8	22.7	22	19.8	0.208^c^
Endometriosis	22 *	14.8	11.4	19.8	0.001^c^
UI	16.5	11.9	15.4	15.5	0.111^c^

*BMI* Body mass index, *UI* Unexplained infertility,

G1. Group 1 BMI ≤ 24.9 kg/m^2^, G2. Group 2 BMI 25–29.9 kg/m^2^, G3. Group 3 BMI ≥ 30 kg/m^2^

Values presented as mean ± standard deviation or n (%)

^a^ANOVA test, ^b^Tukey test post hoc, ^c^Qui-square test

* Different group considering *p* < 0.05

**Fig. 2:**
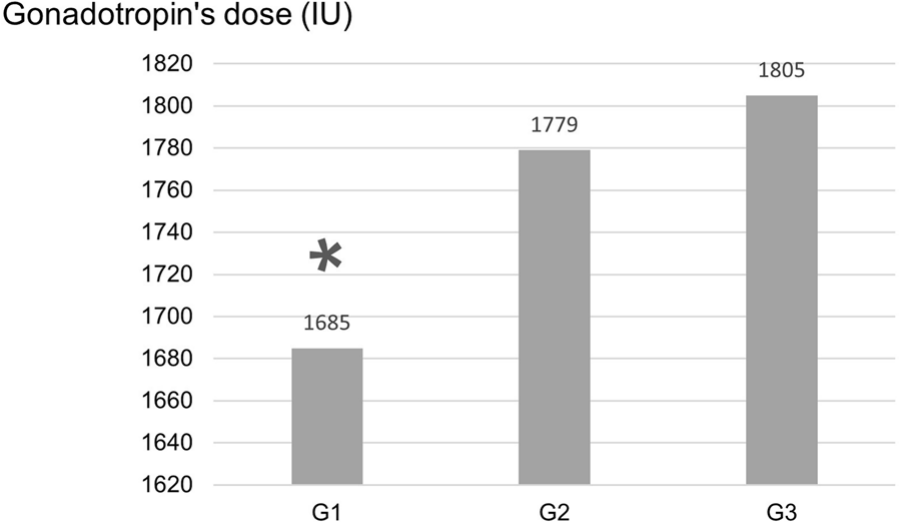
Gonadotropins’ total dose (IU) comparison between groups divided by BMI. G1. Group 1 BMI ≤ 24.9 kg/m^2^, G2. Group 2 BMI 25–29.9 kg/m^2^, G3. Group 3 BMI ≥ 30 kg/m^2^. IU. International units. Values presented as mean obtained by ANOVA and Tukey test post hoc. * *p* = 0.001, when comparing groups

Regarding controlled ovarian stimulation, the laboratory outcomes compared between groups are presented in Table 2. The use of hMG was higher in group 3 (43.6%, *p* = 0.017), while recombinant FSH (FSHr) and corifollitropin alfa (CFA), analyzed separately, were more frequent in group 1 (72.5% FSHr, *p* = 0.046 and 9.3% CFA, *p* = 0.024). No differences were found regarding GnRH protocol chosen or triggering. Gonadotropin’s total doses (IU) when comparing groups 1, 2 and 3 were, respectively: 1685 ± 595 vs. 1779 ± 610 vs. 1805 ± 563 (p = 0.001). Significant higher gonadotropin doses were necessary for groups 2 and 3 responses, with no significant difference between both, as shown in Fig. 2.

**Table 2:** Ovarian stimulation protocol characteristics between groups according to BMI

	G1 *n* = 1940	G2 *n* = 530	G3 *n* = 183	*p*
GnRH protocol (%)				
Agonist	15.8	17.4	15.4	0.673^a^
Antagonist	84.2	82.6	84.6	
Gonadotropins (%)				
hMG	36	39.9	43.6 *	0.017^a^
FSHr	72.5	70.9	64 *	0.046^a^
FSHr +hMG	26.7	31.2	27.4	0.119^a^
FSHr +LHr	7.4	6.9	9.1	0.605^a^
CFA	9.3*	6.9	4.3	0.024^a^
Trigger (%)				
hCGr	88.2	91.7	90.1	0.067^a^
GnRH agonist	11.8	8.3	9.9	

*GnRH* Gonadotropin releasing hormone, *hMG* Urinary gonadotropin, *FSHr* Recombinant Follicle-Stimulating Hormone, *LHr* Recombinant Luteinizing Hormone, *CFA* Corifollitropin alfa, *hCGr* Recombinant Human chorionic gonadotropin

G1. Group 1 BMI ≤ 24.9 kg/m^2^, G2. Group 2 BMI 25–29.9 kg/m^2^, G3. Group 3 BMI ≥ 30 kg/m^2^

Values presented as n (%). * Different group considering *p* < 0.05

^a^Qui-square test and post hoc curve

The ovarian stimulation response analysis is presented in Table 3. There was a lower number of total retrieved oocytes (6 [6–8.7], *p* = 0.014) and retrieved mature oocytes (4 [4.6–6.7], *p* = 0.011) in group 3. An analysis grouping groups 2 and 3 is also shown, with a significantly higher number of total retrieved oocytes (9 [9.4–10.6], *p* = 0.006) found in group 1.

**Table 3:** Ovarian response after stimulation between groups

Variables	G1	G2	G3	*p*
	*n* = 1332	*n* = 373	*n* = 130	
OO	8 [8.4–9]	7 [7.5–8.7]	6 [6–8.7]*	0.014^a^
MII	6 [6.4–7]	6 [5.6–6.6]	4 [4.6–6.7] *	0.011^a^
MII rate (%)	80 [76–78.6]	80 [74.3–79.7]	77.7 [71.4–81]	0.877^a^
	G1	G2 + G3		
OO	9 [9.4–10.6]	8 [8.5–10.1]		0.006^a^
MII	6 [6.9–7.8]	6 [6.3–7.5]		0.333^a^
MII rate (%)	75 [73.8–77.3]	76.9 [72.1–77.7]		0.642^a^

*OO* Total of retrieved oocytes, *MII* Mature retrieved oocytes

G1. Group 1 BMI ≤ 24.9 kg/m^2^, G2. Group 2 BMI 25–29.9 kg/m^2^, G3. Group 3 BMI ≥ 30 kg/m^2^

Values presented as median [interquartile range] or median n (%) [interquartile range]

Adjustment regarding infertility causes was made

^a^ Generalized Estimating Equations

* Statistical different group considering *p* < 0.05

Regarding other laboratory and clinical outcomes after ART analysis (Table 4), fertilization rates were similar between groups, with lower but not statistically significant implantation and pregnancy rates in groups 2 and 3. The embryo development stage at ET was considered for pregnancy rates analysis, and no significant but higher biochemical and clinical pregnancy rates were found when a blastocyst stage (D5) ET was performed. Considering all ET cycles from each patient, it was observed that as higher was the BMI, worse was the cumulative clinical pregnancy rate, with a significant linear tendency (*p* = 0.042).

**Table 4:** ART outcomes analysis compared between groups

Variables	G1	G2	G3	*p*
	*n* = 1270	*n* = 356	*n* = 127	
Fertilization rate (%)	76.6	74.7	76.5	0.442^b^
Implantation rate (%)	28.4	27.5	23.2	0.187^b^
Biochemical pregnancy rate (%)				
Total	44.5	44.8	39.7	0.555^b^
D3	43.3	43	37.2	0.285^b^
D5	46.7	47.5	42.2	0.285^b^
Cumulative^a^	52.6	52	45.1	0.318^c^
*Cumulative Linear Association*				0.204^d^
Clinical pregnancy rate (%)				
Total	40.1	39.7	32.5	0.262^b^
D3	40.4	37.6	30.2	0.466^b^
D5	41.5	41.5	34.4	0.466^b^
Cumulative^a^	48	46.7	36.3	0.061^c^
*Cumulative Linear Association*				**0.042**^b^

G1. Group 1 BMI ≤ 24.9 kg/m^2^, G2. Group 2 BMI 25–29.9 kg/m^2^, G3. Group 3 BMI ≥ 30 kg/m^2^

n. Sample size regarding follicle-stimulation cycles, D3. Embryo transfer in cleavage stage,

D5. Embryo transfer in blastocyst stage

Values presented as n (%). * *p* < 0.05

^a^ Clinical pregnancy among total cycles of each patient

^b^Generalized Estimating Equations, ^c^Qui-square test/post hoc, ^d^Linear-by-linear association

The treatment costs’ analysis and cancellation cycles’ rate are shown in Table 5. A significant linear tendency to higher proportion of canceled IVF cycles as higher the BMI was observed (*p* = 0.001), even considering that group 1 presented the largest sample. When comparing groups 1, 2 and 3, the cancellation cycles’ rate was found to be, respectively: 6.9% vs. 7.8% vs. 10.4% (*p* = 0.002). When cycles were not canceled, gonadotropins total dose performed was lower in group 1 (1685 UI, *p* = 0.001) and, hence, lower treatment costs were associated (U$ 683.02, *p* = 0.001).

**Table 5:** Assisted Reproduction Treatment costs’ analysis

	G1 *n* = 1940	G2 *n* = 530	G3 *n* = 183	*p*
Canceled follicle-stimulation cycles (%)	6.9	7.8	10.4*	0.002^a^ 0.001^2^
Gonadotropins total dose (UI)	1685*	1779	1885	0.001^a^
Gonadotropins total costs (U$)	683.02*	721.13	764.09	0.001^a^

G1. Group 1 BMI ≤ 24.9 kg/m^2^, G2. Group 2 BMI 25–29.9 kg/m^2^, G3. Group 3 BMI ≥ 30 kg/m^2^

^a^Qui-square test and post hoc curve

* Different group considering *p* < 0.05

All data analysis performed in this study for each variable of interest did not consider cycles with missing data information from medical records.

## Discussion

According to this study’s hypothesis, and considering the proposed objective, the results found suggest that overweight and obesity does have a negative impact on laboratory and clinical ART outcomes.

The mean maternal age was clinically similar between groups, but a significant difference was found, probably regarding the unequal sample size. As shown in Table 1 among the infertility causes, male factor infertility was less prevalent in overweight and obese groups, while endometriosis was significantly higher in eutrophic women. No differences were found regarding other infertility causes. It is important to highlight that each infertile couple could have presented more than one infertility cause.

Regarding ovarian controlled stimulation, the study’s findings on gonadotropins’ doses (Table 2) have agreed with other studies, showing increased gonadotropin doses needed for better ovarian stimulation response in both overweight and obese patients. The higher the BMI, higher was the gonadotropin mean dose needed, even with a limited sample size. This result reinforces the poor ovarian response expected not only for obese, but also for overweight women.

Furthermore, it is possible that the higher proportion of canceled IVF cycles, more frequently found in overweight and obesity patients, was related to the same poor ovarian response that required higher gonadotropins doses. This need of increased doses requires, hence, higher treatment costs and the probability of more than one cycle for achieving pregnancy, which could be the reason why more overweight and obese women discontinued cycles initiated. Kawwas et al. [19] analyzed only the first autologous fresh cycle from each patient undergoing ART and showed higher chances of canceled cycles in obese patiens compared to eutrophic women, in agreement to our findings, as well as the clinical trial from Roth et al. [8], in which increased gonadotropin doses and canceled cycles rates were once again related to obese patients. Moreover, Zhou et al. [6] retrospectively analyzed the first IVF-ET cycle from patients undergoing assisted reproduction and showed higher gonadotropin doses in overweight and obese; differently from other studies, however, they found a low cycle cancelation rate in these patients [20].

The low number of retrieved and mature oocytes found in overweight and obese patients, as demonstrated in Table 4, followed the expectation that higher BMI is related to worse ovarian response, even with a similar oocyte maturity rate. This finding is in agreement with data from other studies, as reviewed by Amiri & Ramezani Tehrani [9]. The analysis putting groups 2 and 3 together suggested that being overweight is enough to worsen the ovarian response. Zhou et al. [6] also found better number of retrieved oocytes in eutrophic women when compared to overweight and obese patients, and the overweight also enough for this negative impact to be found. Moragianni et al. [21], divided patients in six groups according to BMI, from underweight to obese patients’ class III, and analyzed only fresh IVF cycles, finding, as well as our study, worse ovarian response in overweight and obese patients; different from our results, however, no differences were found in cycle cancellation rate or gonadotropin doses for ovarian stimulation.

Even with higher gonadotropin doses performed in overweight and obese patients for achieving better ovarian response, this was not translated into better total number of retrieved oocytes and mature. However, the significant difference was not maintained for oocyte maturity rate, with low but not statistically different in the obese group.

The canceled IVF cycles found in our study seems to be, thus, related to worsen controlled ovarian stimulation found in overweight and obese women. Those obese patients who did not discontinue cycles and had the oocyte retrieval performed were probably women with a similar hormonal profile than eutrophic, despite BMI. This could be the explanation for the lack of significant difference found in oocyte maturity rate.

Regarding clinical outcomes, the laboratory outcomes’ results were not translated into fertilization rates differences, as shown in Table 4. In the systematic review from Amiri & Ramezani Tehrani [9], some of the studies analyzed presented worse fertilization rates, while others, like ours, did not. The similar fertilization rates found in this present study could be related to the ART advances seen in the last decade and should not be seen as a good oocyte quality marker in these cases, as even higher fertilization rates were not translated into better implantation, pregnancy and live birth rates (LBR), all found to be worse in overweight and obese women. Although some of these differences found were not statistically significant, they are clinically relevant.

Biochemical and clinical pregnancy rates tended to be higher when a blastocyst ET was performed in eutrophic, overweight and obese women (Table 4 This finding agrees with previous studies showing better clinical outcomes when comparing blastocyst ET to cleavage stage [22]. Therefore, to improve pregnancy rates observed in obese patients, the transfer of a blastocyst stage embryo could be prioritized in the impossibility of patient’s weight loss before ART.

When cumulative pregnancy rates were analyzed, the significant linear association found with pregnancy rates being inversely proportional with the BMI suggests that weight excess negatively impacts clinical outcomes even when considering all the cycles each patient went through. This finding corroborates with Amiri & Ramezani Tehrani [9] conclusion of major difficulty to achieve spontaneous pregnancy in obese women even when they presented a regular ovulation. Therefore, obese patients in reproductive treatment are expected to present an even higher time taken to conceive, despite the number of follicle-stimulation cycles performed.

When considering treatment costs, the higher was the BMI the higher was the cancelation cycles rate and the gonadotropin doses needed to achieve a similar ovarian response. This relation leaded, consequentely, to a more expensive ART treatment for overweight and obese patients seeking pregnancy, in agreement with Koning et al. [14] and Denison et al. [15] studies.

### Study’s limitations

An important highlight to be mentioned is the BMI’s low reflection of actual body fat percentage, despite being a relevant populational classification. The abdominal circumference (AC) measure was demonstrated by Christofolini et al. [23] to be a more accurate measure than BMI to predict unfavorable ART outcomes, in agreement with Ryan & Kahan [24] American Guidelines Recommendations on its importance for better weight evaluation. Thus, AC could be implemented in assisted reproduction centers and more studies comparing its effect with BMI should be performed regarding assisted reproduction.

Furthermore, the limited sample size in this study was an important limitation to statistical power analysis, leading to some non-significant results found to be still considered as clinically relevant for patients in reproductive treatment. Studies considering more cycles could achieve 80% power and find statistical significant differences.

As a retrospective study, limitations regarding data collection were considered. Analysis of other ovarian function markers, such as FSH, LH and AMH, were not included due to lack of information on medical records. More studies including this marker could bring better conclusions.

## Conclusions

In conclusion, overweight and obesity are associated with worse clinical outcomes in assisted reproduction, with worse ovarian response even using higher gonadotropin doses for stimulus and lower chances of implantation and clinical pregnancy rates.

These findings also highlight the importance of considering the higher treatment costs for overweight and obese patients, beyond all the well-known risks regarding weight excess, fertility, and pregnancy, before starting assisted reproduction treatments.

It is important to reinforce that, regardless of assisted reproduction, as obese pregnant women are at well-established increased risk for maternal, perinatal, and fetal complications, the benefit of pre conceptional maternal weight loss should be widely recommended for a benefit before and during pregnancy.

## Data Availability

The datasets generated and analyzed in the present study are not publicly available due to patient’s confidentiality; however, on reasonable request, they are all available with the corresponding author.

## Abbreviations

BMI: Body Mass Index
ART: Assisted Reproductive Techniques
LBR: Live Birth Rate
ET: Embryo Transfers
IVF: In Vitro Fertilization
GnRH: Gonadotropin-Releasing Hormone
ICSI: Intracytoplasmic Sperm Injection
hCG: Human Chorionic Gonadotropin
SPSS: Social Package for Social Sciences
GEE: Generalized Estimating Equations
